# Assessing Knowledge, Attitudes, and Practices (KAP) Regarding Organ Donation Among the General Population in Perambalur, India: A Cross-Sectional Study

**DOI:** 10.7759/cureus.80410

**Published:** 2025-03-11

**Authors:** S Vijayalakshmi, Ramkumar Sundaram, Shagirunisha Rizvana, Aswin A, Srividhya S, Sriram Sooriya, Suriya Khumar, Sunitha R, Subashini V, Sujitha G

**Affiliations:** 1 Community Medicine, Dhanalakshmi Srinivasan Medical College and Hospital, Perambalur, IND; 2 Community Medicine, Sree Mookambika Institute of Medical Sciences, Kulasekharam, IND

**Keywords:** community-based study, cross-sectional studies, epidemiology, organ donation, public awareness

## Abstract

Background

Organ transplantation is a successful medical intervention available for end-stage organ failure. There is a wide gap between the need and the availability of organs.The government has begun specific, long-term initiatives to encourage organ donation, although it has not been able to come to a stage of full realisation.

Objective

The objective of this study is to assess the knowledge, attitude, and practice (KAP) regarding organ donation among the general population in Perambalur, India.

Methodology

This cross-sectional study was performed among the general population in Perambalur, Tamil Nadu, India, from December 2022 to February 2023. About 470 individuals were selected using the convenience sampling method. A semi-structured pro forma was used to collect the socio-demographic profile, and a questionnaire was used to assess the KAP of organ donation. The minimum and maximum scores for knowledge of organ donation were 0-13 and for attitude and practice as 0-8. The data were analyzed using Statistical Package for the Social Sciences (IBM SPSS Statistics for Windows, IBM Corp., Version 26.0, Armonk, NY), and the categorical data were represented as frequency and percentage, whereas mean and standard deviation represent quantitative data. The chi-square test was used to investigate the relationship between sociodemographic characteristics and KAP regarding organ donation.

Results

A total of 470 responses were analyzed, with 57.7% males and 65.3% from rural areas. Most (84.7%) had heard of organ donation, mainly through television (78.6%). Awareness was highest for eye (89.4%), kidney (76%), and heart (71%) donation. While 83.6% supported organ donation, only 16.8% pledged, and 5% registered as donors. Adequate KAPs were seen in 34.1%, 29.1%, and 27.1% of participants, respectively. Younger age, urban residence, higher education, and upper socioeconomic class were significantly associated with better KAP scores (p=0.001).

Conclusion

Despite high awareness and a positive attitude toward organ donation, actual donor registration remains low. More than one-fourth of the participants had adequate KAP, influenced by factors such as younger age, urban residence, higher education, and upper socioeconomic status. Misconceptions, cultural beliefs, and religious concerns were key barriers to participation. Targeted awareness campaigns involving media, healthcare professionals, and religious scholars are crucial to increasing acceptability. Strengthening donor registration systems and policy-driven incentives can help bridge the gap between awareness and actual organ donation.

## Introduction

Organ donation is the removal of tissue from the human body (living/dead) for transplantation into another person as a treatment. Organs that can be donated and transplanted include kidneys, hearts, livers, pancreas, intestines, lungs, skin, bone marrow, cornea, etc. [1]. End-stage organ failure can be successfully treated by organ transplantation. Due to a lack of suitable donor organs, organ donation has become an increasingly important public health issue over the past few decades. It is a major factor in medical, reproductive, and transplant tourism around the world when combined with other demands. Organ donors are currently divided into two categories: donor after brain death and donor after cardiac death, with organ/tissue removal feasible in both the live and deceased states. Organ and tissue transplantation is a costly medical intervention that is predominantly driven by the country’s private sectors, and young deaths as a result of a vehicle accident or a cardiovascular catastrophe provide the best option for high-quality organ yield. To date, no country in the world has collected enough organs to meet the requirements of its population [2-4].

The scarcity of donor organs is the fundamental impediment to a successful deceased donor organ transplantation programme. The cadaver organ donation programme in India is still in its infancy, hampered by a lack of proper information among doctors regarding the criteria and standards for declaring brain dead. Although the public is used to the idea of donating blood, organ donation after death remains a challenge. There is an urgent need to raise public knowledge regarding organ transplantation and donation. Increasing the donor pool is a critical public health concern. One factor that could contribute to the scarcity of donor organs is a lack of information about the legal and procedural aspects of organ donation [3,5-7].

The Transplantation of Human Organs Act of 1994 regulates the removal, storage, and transplantation of human organs for therapeutic purposes, prohibits the sale of human organs for profit, and addresses related ethical concerns. However, even 20 years after its enactment, cadaver donations remain sporadic, while live kidney donations are more common [8]. The demand for organ transplants in India far exceeds the supply. An estimated 1.8 million people experience renal failure annually, yet only about 6,000 kidney transplants are performed. A timely liver transplant could save 10% to 15% of the 200,000 people who die each year from liver disease or cancer, but only about 1,500 liver transplants are conducted. Similarly, while over 50,000 individuals die from heart failure each year in India, only 10 to 15 heart transplants are performed. For cornea transplants, the need stands at approximately 100,000 annually, but only around 25,000 procedures are carried out [9].

In addition to organ failure statistics, studies have highlighted significant disparities in organ donation awareness and willingness to donate across different demographics in India. A study by Bapat and Kedlaya [3] found that only 30% of surveyed individuals were aware of brain death as a legal criterion for organ donation. Similarly, a survey conducted in South India [10] reported that religious beliefs and misconceptions about organ retrieval procedures significantly impacted the decision to donate. Addressing these gaps through targeted interventions is crucial for improving donation rates.

To bridge this gap, the government has launched ongoing initiatives to promote organ and tissue donation, but it has yet to achieve full reality. This situation has several potential causes, including problems with the individual, the community, and the health system [2]. Therefore, this study aims to assess the awareness and practice of organ donation among the general population in Perambalur, India. The findings will contribute to the existing literature and provide valuable insights for healthcare providers and policymakers in developing effective interventions, such as media-based education campaigns, legislative amendments, and community outreach programs, to improve organ donation rates.

## Materials and methods

Study design and study setting

This cross-sectional study was conducted among the general population of Perambalur, Tamil Nadu, India.

Study period

The study was conducted over a period of three months from December 2022 to February 2023.

Ethics committee approval

The study protocol was approved by the Institutional Ethics Committee of Dhanalakshmi Srinivasan Medical College and Hospital, Perambalur (approval number: IECHS/IRCHS/No. 290). Written informed consent was obtained from all participants after explaining the study objectives and procedures. The confidentiality and anonymity of the participants were maintained throughout the study.

Inclusion criteria

Adults aged 18 years and above, permanent residents of Perambalur district for at least six months, and those willing to participate in the study were included. Both male and female participants who could comprehend and respond to the questionnaire were eligible for participation.

Exclusion criteria

Individuals with diagnosed psychiatric disorders, shift workers, and those unable to provide informed consent were excluded from the study. Participants with incomplete questionnaire responses were also excluded from the final analysis.

Sample size and sampling technique

The sample size was calculated considering a study by Bharambe et al. showing 43.9% were willing to be organ donors in an urban city [10] in India and after applying the formula, $n = \frac{Z^2_{1-\alpha/2} \cdot p \cdot q}{d^2}$ (Z^2^_1-α/2_=1.96, p=43.9, q=100-p(43.9), d=5), the sample size came up to 378. The study collected data from 470 subjects by convenience sampling. The questionnaire was pretested on 10 subjects who were later excluded from the study and analysis.

Sampling method

The study utilized a convenience sampling technique for participant recruitment, acknowledging both methodological considerations and practical constraints of community-based research. The sampling process was executed in two distinct phases. Initially, the district was stratified into urban and rural areas to ensure representation from both settings, and accessible areas within each stratum were identified based on logistical feasibility and population density. Subsequently, eligible participants were recruited through consecutive sampling from the identified areas based on their availability and willingness to participate. To minimize selection bias, recruitment was conducted at varying times of the day and different days of the week, continuing until the calculated sample size was achieved.

Data collection procedure

Data collection was conducted by trained research assistants through face-to-face interviews using a structured questionnaire. The questionnaire was initially developed in English and translated to the local language following standard forward-backward translation procedures. A pilot study was conducted among 10 participants (not included in the final analysis) to assess the feasibility and reliability of the questionnaire. Based on the pilot study feedback, necessary modifications were made to improve clarity and comprehension.

Study tool

The questionnaire contains five parts: sociodemographic profile, awareness, knowledge, attitude, and practice towards organ donation. The sociodemographic details included age, gender, residence, occupation, religion, family members, education qualification, and monthly income. The socioeconomic status was classified according to the modified BG Prasad scale [11]. The second section collected information about awareness of organ donation. The third and fourth sections included 13 basic knowledge questions and eight attitude questions about organ donation. The final section included eight practice questions about organ donation. The answer to every positive item was coded as Yes=1, No=0, and reversely coded for negative questions. The minimum and maximum scores for knowledge of organ donation were 0-13, and for attitude and practice were 0-8, respectively. The participants who scored ≥75 percentile as good KAP (knowledge ≥8, attitude ≥7, practice ≥1) and who scored less than 75 percentile as poor KAP.

Data analysis

The data collected was entered into Microsoft Excel (Microsoft® Corp., Redmond, WA, USA) and analyzed using Statistical Package for Social Sciences (SPSS) version 26.0 (IBM Corp., Armonk, NY). The descriptive analysis was analyzed using frequencies and percentages for categorical variables, while continuous variables were expressed as mean ± standard deviation. The chi-square test was used to assess the association between categorical variables, with p<0.05 considered statistically significant. Missing data were handled using multiple imputation techniques to minimize bias and ensure robust findings.

## Results

Table 1 shows the sociodemographic and personal characteristics of study participants. A total of 470 responses were obtained during the study duration. The majority of participants (271, 57.7%) were males, and 181 (38.5%) belonged to the age group of 31 to 45 years. The participants were from Perambalur, of which 307 (65.3%) comprised rural areas. A total of 67.7% of subjects had a middle-school education qualification. The majority of the population (71.7%) belongs to the Hindu religion. More than half of the participants (53.2%) belong to a socioeconomic class I or II, according to BG Prasad’s modified Classification for 2022.

**Table 1:** Sociodemographic and personal characteristics of study participants (n=470)

Characteristics		n (%)
Age (in years)	≤30	86 (18.3)
	31 to 45	181 (38.5)
	46 to 60	133 (28.3)
	61 to 75	59 (12.6)
	>75	11 (2.3)
Sex	Male	271 (57.7)
	Female	199 (42.3)
Religion	Hindu	337 (71.7)
	Muslim	62 (13.2)
	Christian	71 (15.1)
Residence	Urban	163 (34.7)
	Rural	307 (65.3)
Education	Illiterate	95 (20.2)
	Up to 5^th^ class	57 (12.1)
	6^th^ to 12^th^ class	147 (31.3)
	Undergraduate degree	135 (28.7)
	Postgraduate degree	36 (7.7)
Occupation	Unemployed	3 (0.6)
	Unskilled	51 (10.9)
	Semiskilled	228 (48.5)
	Skilled	37 (7.9)
	Arithmetic skilled jobs	74 (15.7)
	Semi-professional	9 (1.9)
	Professional	68 (14.5)
Socioeconomic class	Class I	123 (26.2)
	Class II	127 (27)
	Class III	83 (17.7)
	Class IV	102 (21.7)
	Class V	35 (7.4)

Knowledge

**Figure 1:**
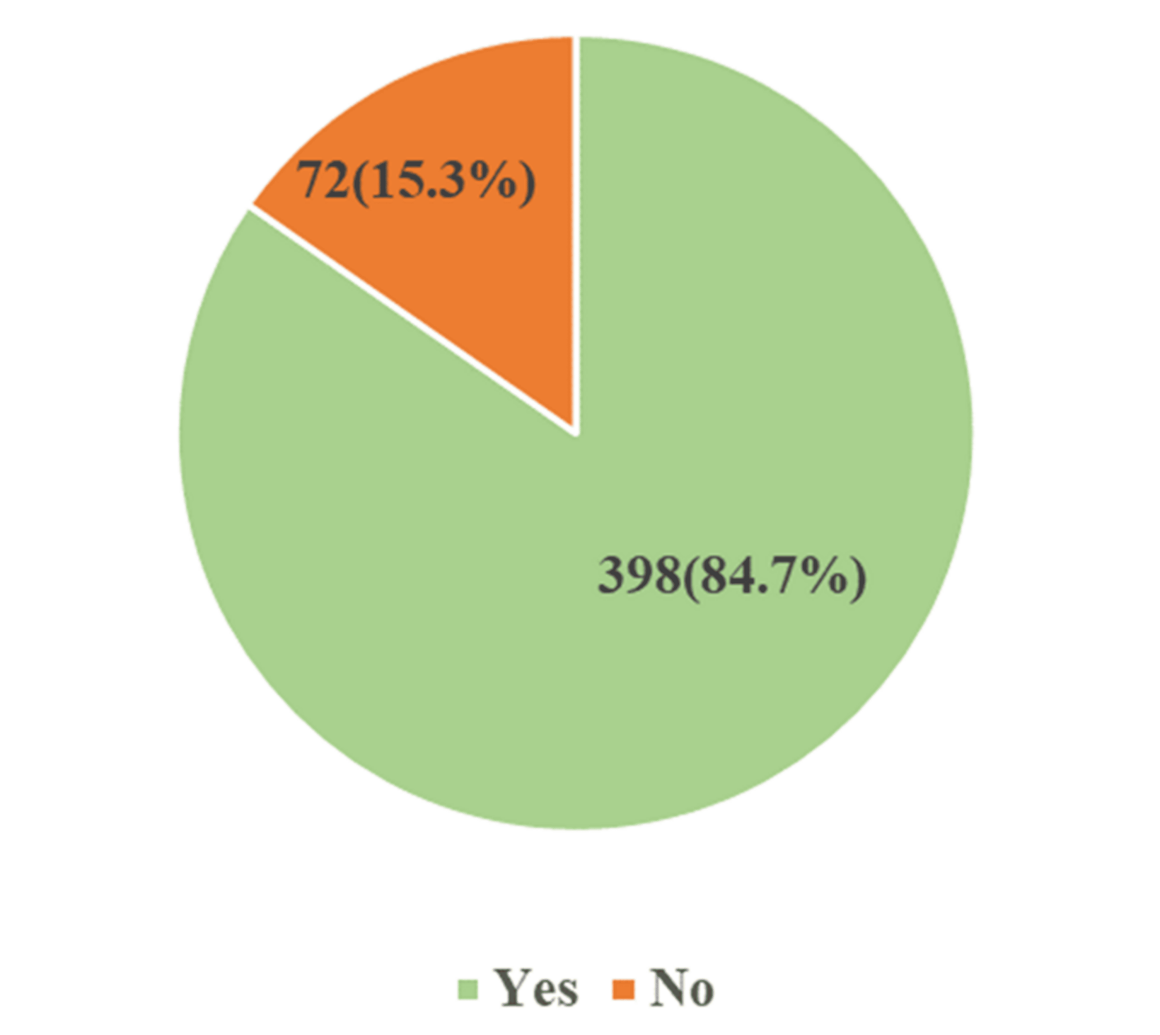
Awareness of organ donation among study participants (n=470)

Figure 1 shows the awareness of organ donation among study participants. Out of 470 respondents, 398 (84.7%) had heard the term "Organ Donation." The mean ± SD for knowledge was 6.47 ± 2.23. Participants who had not heard of the term organ donation were not asked further questions about the study. A total of 78.6% of the participants consider television as their source of knowledge for organ donation, 38.8% from the newspaper, 37.8% from a doctor, 36.8% from the internet, 35% from radio, and 24.4% from friends. According to this study, most of the participants were aware of donating eyes - 356 (89.4%), kidneys - 304 (76%), and hearts - 286 (71%).

Table 2 shows the knowledge regarding organ donation among participants. Among the respondents, only 29.4% knew where to obtain an organ donation card, while a significant (85.2%) were aware that some people die due to the non-availability of organs. A majority (70.1%) believed that people in society do donate organs, and 58.8% had heard of brain death. However, only 52.8% were aware that brain-dead patients could donate organs. Knowledge about the Transplantation of Human Organs Act was limited, with only 32.9% of participants being aware of it. Additionally, 26.1% incorrectly believed that organs could be removed without patient or relative consent. Concerns about organ misuse were minimal, as 81.2% believed that donated organs were not misused or abused. Regarding decision-making in organ donation, 54.3% acknowledged that parents or guardians could make substitute decisions for mentally disabled individuals. Furthermore, 57.7% knew from whom consent should be obtained for living organ donations, while only 6.4% were aware of who should decide in the case of an unclaimed dead body. Among married individuals, 31.9% knew from whom to obtain consent for organ donation after death.

**Table 2:** Knowledge regarding organ donation among participants (n=398)

Knowledge	Yes n (%)	No n (%)
Knows where to obtain an organ donation card	117 (29.4)	281 (70.6)
Aware that organ shortage causes deaths	339 (85.2)	59 (14.8)
Believes organ donation happens in society	279 (70.1)	119 (29.9)
Have you heard of brain death	234 (58.8)	164 (41.2)
Knows brain-dead patients can donate organs	210 (52.8)	188 (47.2)
Familiar with the Transplantation of Human Organs Act	131 (32.9)	267 (67.1)
Believes organs can be removed without consent	104 (26.1)	294 (73.9)
Thinks donated organs are misused or abused	75 (18.8)	323 (81.2)
Parents/guardians make substitute decision-making for mentally disabled persons after death regarding organ donation	216 (54.3)	182 (45.7)
Knows when an organ can be donated	228 (57.2)	170 (42.7)
Aware of consent rules for living organ donation	271 (57.7)	127 (42.3)
Knows who decides organ donation for an unclaimed body	30 (7.5)	368 (92.5)
Knows who gives consent for donation in married individuals	150 (37.7)	248 (62.3)

Attitude

Table 3 shows the attitude towards organ donation. The majority of participants (83.7%) supported organ donation, and 79.9% felt comfortable discussing it. Regarding religious beliefs, 68.3% believed their religion supported organ donation. More than half (57.3%) were willing to sign a pledge card for organ donation. When asked about receiving organs from individuals of different religions or families, 78.6% of participants were accepted in both cases. However, 47.5% stated they would donate their organs only to their family members. Additionally, 77.1% believed there should be a monetary incentive for organ donation. The mean ± SD for attitude was 5.22 ± 2.09.

**Table 3:** Attitude toward organ donation among study participants (n=398)

Attitude	Yes n (%)	No n (%)
Supports organ donation	333 (83.7)	65 (16.3)
Feels comfortable discussing organ donation	318 (79.9)	80 (20.1)
Believes their religion supports organ donation	272 (68.3)	126 (31.7)
Willing to sign an organ donation pledge card	228 (57.3)	170 (42.7)
Accepts an organ from a donor of a different religion	313 (78.6)	85 (21.4)
Accepts an organ from a donor of a different family	313 (78.6)	85 (21.4)
Would donate organs only to family members	189 (47.5)	209 (52.5)
Believes monetary incentives should be given for organ donation	307 (77.1)	91 (22.9)

Practice

Despite a positive attitude toward organ donation, actual practice was significantly low. Only 16.8% had planned to donate their organs, and a mere 5.0% had signed up to donate. Furthermore, only 3.0% had donated an organ, and 4.0% had received one. Among family members, 4.5% had pledged to donate their organs, while 5.3% had signed up for organ donation. Additionally, 6.8% had a family member who received an organ, while 5.3% had a family member who had previously donated an organ. Among 398 participants, only 67 (16.8%) pledged to donate their organs, out of which 20 (5%) signed up to donate organs. The mean ± SD for practice was 0.51 ± 1.13 (Table 4).

**Table 4:** Practice of organ donation among the study participants (n=398)

Practice	Yes n (%)	No n (%)
Has planned to donate their organs	67 (16.8)	331 (83.2)
Has officially signed up for organ donation	20 (5.0)	378 (95.0)
Has personally donated an organ	12 (3.0)	386 (97.0)
Has received an organ transplant	16 (4.0)	382 (96.0)
Has a family member who pledged to donate	18 (4.5)	380 (95.5)
Has a family member who signed up for organ donation	21 (5.3)	377 (94.7)
Has a family member who received an organ transplant	27 (6.8)	371 (93.2)
Has a family member who donated an organ before	21 (5.3)	377 (94.7)

Knowledge, attitude, and practice (KAP)

**Figure 2:**
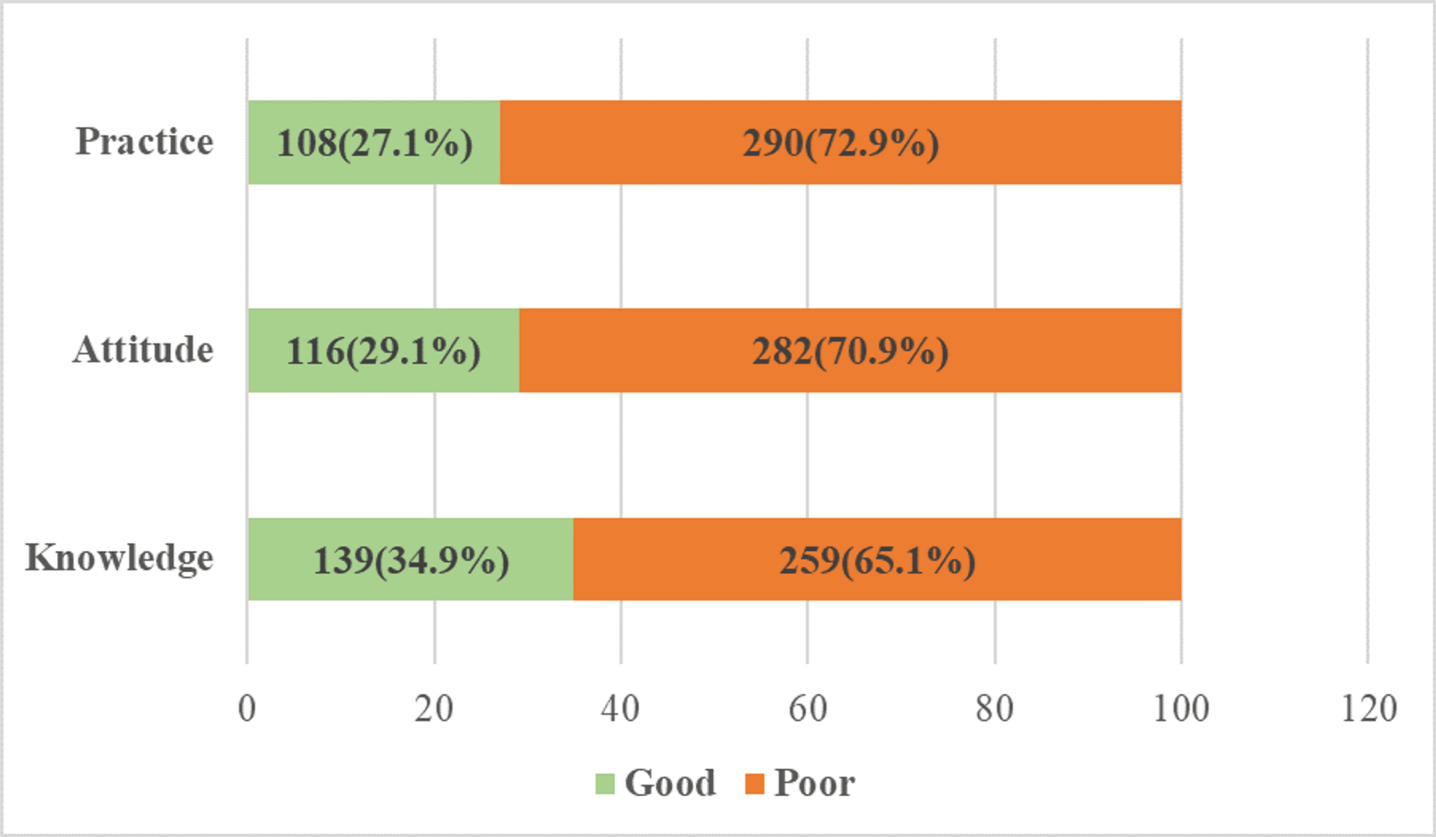
Level of knowledge, attitude, and practice on organ donation among study participants (n=398)

Among the study participants, 139 (34.1%), 116 (29.1%), and 108 (27.1%) had adequate KAP regarding organ donation (Figure 2).

Table 5 shows the association between sociodemographic profile and KAP regarding organ donation. Participants who were younger than 40 years had adequate knowledge (83, 42.6%), attitude (62, 31.8%), and practice (59, 30.3%) on organ donation when compared to their counterparts and knowledge, which is statistically significant (p=0.002). Participants who were following Islam had adequate knowledge (27, 47.4%) and practice (28, 49.1%), which is statistically significant, p=0.012, p<0.001, respectively. Participants who were residing in urban areas studied graduate education and semi-professionals or professionals had adequate knowledge and practice on organ donation which is statistically significant (p<0.001). Participants who belong to the upper class had adequate KAP, which is statistically significant (p<0.001).

**Table 5:** Association between basic characteristics and levels of KAP on organ donation among study participants (n=398) * The chi-square test was used and statistically significant when the p-value was less than 0.05. KAP: knowledge, attitude, and practice

Variables		Knowledge		p-value	Attitude		p-value	Practice		p-value
		Good n (%)	Poor n (%)		Good n (%)	Poor n (%)		Good n (%)	Poor n (%)	
Age (in years)	≤40	83 (42.6)	112 (57.4)	0.002*	62 (31.8)	133 (68.2)	0.254	59 (30.3)	136 (69.7)	0.170
	>40	56 (27.6)	147 (72.4)		54 (26.6)	149 (73.4)		49 (24.1)	154 (75.9)	
Sex	Male	76 (31.8)	163 (68.2)	0.109	69 (28.9)	170 (71.1)	0.882	72 (30.1)	167 (69.9)	0.100
	Female	63 (39.6)	96 (60.4)		47 (29.6)	112 (70.4)		36 (22.6)	123 (77.4)	
Religion	Hindu	85 (30.4)	195 (69.6)	0.012*	88 (31.4)	192 (68.6)	0.251	64 (22.9)	216 (77.1)	<0.001*
	Muslim	27 (47.4)	30 (52.6)		12 (21.1)	45 (78.9)		28 (49.1)	29 (50.9)	
	Christian	27 (44.3)	34 (55.7)		16 (26.2)	45 (73.8)		16 (26.2)	45 (73.8)	
Residence	Urban	88 (55.3)	71 (44.7)	<0.001*	52 (32.7)	107 (67.3)	0.203	62 (39)	97 (61)	<0.001*
	Rural	51 (21.3)	188 (78.7)		64 (26.8)	175 (73.2)		46 (19.2)	193 (80.8)	
Education	Illiterate	5 (9.3)	49 (90.7)	<0.001*	10 (18.5)	44 (81.5)	0.126	7 (13)	47 (87)	<0.001*
	Primary	2 (5.3)	36 (94.7)		9 (23.7)	29 (76.3)		5 (13.2)	33 (86.8)	
	Secondary	39 (28.5)	98 (71.5)		39 (28.5)	98 (71.5)		34 (24.8)	103 (75.2)	
	Graduate	93 (55)	76 (45)		58 (34.3)	111 (65.7)		62 (36.7)	107 (63.3)	
Occupation	Unemployed/unskilled	7 (14.9)	40 (85.1)	<0.001*	7 (14.9)	40 (85.1)	0.065	9 (19.1)	38 (80.9)	0.009*
	Semiskilled/skilled	77 (28)	198 (72)		84 (30.5)	191 (69.5)		68 (24.7)	207 (75.3)	
	Semi-professional/professional	55 (72.4)	21 (27.6)		25 (32.9)	51 (67.1)		31 (40.8)	45 (59.2)	
Socioeconomic class	Class I	78 (63.9)	44 (36.1)	<0.001*	39 (32)	83 (68)	<0.001*	48 (39.3)	74 (60.7)	<0.001*
	Class II	39 (32.8)	80 (67.2)		49 (41.2)	70 (58.8)		33 (27.7)	86 (72.3)	
	Class III	15 (22.1)	53 (77.9)		17 (25%)	51 (75)		15 (22.1)	53 (77.9)	
	Class IV	3 (4.2)	68 (95.8)		8 (11.3%)	63 (88.7)		10 (14.1)	61 (85.9)	
	Class V	4 (22.2)	14 (77.8)		3 (16.7%)	15 (83.3)		2 (11.1)	16 (88.9)	

## Discussion

This cross-sectional study was conducted to assess the KAP regarding organ donation among the general population in Perambalur, India. In our study, 84.7% of the population was aware of the term organ donation. A study conducted in Puducherry by Balajee et al. [7] showed a similar awareness level of 88%, whereas Manojan et al. [12] in Kerala reported an even higher awareness rate of 97%. These differences can be attributed to regional variations and differing literacy levels among the population.

Regarding sources of awareness, our study found that 78.6% of participants became aware of organ donation through television. In contrast, a study conducted in western India [6] indicated that 48% of people heard about organ donation through the medical fraternity. This highlights the strong influence of media in disseminating information about organ donation.

Concerns about organ misuse were also prevalent. Our study showed that 64.1% of aware participants feared potential misuse of donated organs, which aligns with findings from a study in western India [6], where 59% of respondents held similar concerns. This fear may stem from misinformation and a lack of education regarding the organ donation process.

Awareness of legal aspects was limited in our study, with only 32.9% of participants aware of the Organ Transplantation Act. In comparison, a study among final-year students in Chennai [13] found that 54% were aware of the act, indicating that legal awareness is significantly lower among the general population. Similarly, 70.7% of our study participants did not know where to obtain an organ donation card, compared to 43% of students in Punjab [14] lacking this knowledge. These gaps emphasize the need for targeted awareness campaigns.

The overall KAP score for organ donation in our study was approximately 30%. Factors associated with adequate knowledge included age ≤ 40 years, whereas urban residence, higher education, and professional occupation were linked to both knowledge and practice. Religious beliefs also played a role, with significant differences observed between religious groups, particularly regarding willingness to donate. A study in Pakistan by Saleem et al. [15] also found that education, socioeconomic status, and religion were significant predictors of organ donation willingness. Future studies should explore these differences in more depth.

Barriers to organ donation, such as misinformation, cultural beliefs, and distrust in the medical system, were also evident. Qualitative insights from future research could further elucidate these factors and help design more effective interventions to address low donor rates.

Strength

The strength section highlights the study’s key advantages, including its adequate sample size (470 participants), ensuring statistical power. The stratified sampling approach covering both urban and rural areas enhances representativeness. The use of a structured, pretested questionnaire ensures consistency, and face-to-face interviews minimize response bias. Research assistants received structured training, and random data quality checks (≥10%) were conducted to enhance reliability. Appropriate statistical methods, such as chi-square tests and multiple imputation techniques for missing data, further strengthened the study's validity. Additionally, ethical transparency was maintained with Institutional Ethics Committee approval and informed consent, ensuring credibility and ethical rigor.

Limitations

This study relied on subjective assessments, limiting the depth of understanding regarding factors affecting organ donation. Conducting the study in a single district restricts generalizability to broader populations. Furthermore, it did not evaluate the impact of specific public health campaigns or interventions on awareness and participation. Self-reported responses may also introduce recall or social desirability bias, potentially affecting the accuracy of the reported KAPs. Future studies should incorporate mixed-method approaches, including qualitative insights, to better understand the barriers and facilitators of organ donation behavior.

## Conclusions

Despite high awareness and a generally positive attitude toward organ donation, actual commitment to donation remains low. More than one-fourth of the study participants had adequate KAP, with younger individuals (≤40 years), urban residents, Muslims, graduates, professionals, and those from upper socioeconomic classes demonstrating better KAP scores. The findings suggest that education, socioeconomic status, and cultural influences play a significant role in shaping perceptions toward organ donation. However, while many participants supported organ donation in principle, only a small fraction had pledged or registered as donors, highlighting a crucial gap between awareness and action. Misinformation, religious concerns, cultural beliefs, and a lack of motivation were identified as major barriers preventing individuals from actively engaging in organ donation.

Bridging this gap requires a multi-pronged approach, including targeted awareness campaigns, community engagement, and policy interventions. Public health initiatives should focus on dispelling myths, addressing religious and cultural apprehensions, and emphasizing the life-saving impact of organ donation. The involvement of media, healthcare professionals, and religious scholars can help build public trust and encourage wider acceptance. Additionally, improving accessibility to donor registration, offering incentives, and integrating organ donation education into community health programs can further enhance participation. By addressing these challenges and promoting evidence-based interventions, organ donation rates can be significantly improved, ultimately saving more lives and meeting the growing demand for organ transplants.

## Appendices

Appendix A

Organ Donation Questionnaire

1.           Age:

2.           Sex:   Male/Female

3.           Religion:   Hindu/Muslim/Christian     Other:

4.           Education: Illiterate/Up to 5th class/From 6th to 12th class/Undergraduate degree/Postgraduate degree/Doctorate/Other:

5.           Occupation:

6.           Residence: Urban/Rural

7.            No of persons in the family:

8.           Total family income per month:

Knowledge

9.               Have you heard of the term organ donation?: Yes/No

10.           You heard about organ donation through which of the following sources: Heard from the doctor/Internet/online resources/Television/Radio/Newspaper or Magazines/Friend/Colleague/Other:

**Table 6:** Knowledge

11.	Yes	No
Do you know where to obtain an organ donation card?		
Do you know that some people die because of non-availability of organs?		
In general, do people in society donate organs?		
Have you heard of brain death?		
Can Brain-dead patients' organs can be donated?		
Are you aware of 'Transplantation of humans organ act'		
Can organs be removed without the permission of the patient / relative?		
Do you feel organs donated are misused /abused?		
Can parents/guardians make substitute decision-making for mentally disabled persons after death regarding organ donation?		

12. Do you know when an organ can be donated: Alive/Dead/Both (alive/dead)/Don't know/Other:                  

13. What organs can be donated? Eye/Lung/Heart/Liver/Kidney/Pancreas/Other:            

14. For living donation, from who do we get consent? Donor/Family members/Spouse/Doctor/Friends/Relatives/Other:      

15. Who should make decisions about organ donation in case of unclaimed dead bodies? Charitable Organisations/Doctor/Police/Judge/None/Don’t know/Others.______

16. For donation after death, from who should we get consent in married person: No one/Family members/Spouse/Doctor/Friends/Other:  

Attitude

17.           Do you support organ donation?  Yes/No 

18.           Do you feel comfortable to talk about organ donation?  Yes/No

19.           Does your religion support organ donation?  Yes/No 

20.           Are you ready to sign a pledge card for organ donation? Yes/No

21.           Is your attitude towards organ donation influenced by others? Yes/No 

22.           Do you accept organs from persons of different religions? Yes/No 

23.           Do you accept organs from people of different families? Yes/No 

24.           Do you donate, your organ only to your family? Yes/No 

25.           Do you think there should be a monetary incentive for organ donation? Yes/No 

Practice

26.           Have you pledged to donate your organs? Yes/No

27.           Have you signed to donate your organ? Yes/No

28.           Have you ever donated an organ? Yes/No

29.           Did you ever receive an organ for transplantation? Yes/No

30.           Have your family members pledged to donate their organs? Yes/No

31.           Have your family members signed up to donate their organs? Yes/No

32.           Did your family members receive an organ for transplantation? Yes/No

33.           Did your family members donate an organ before? Yes/No
